# Experimental and Numerical Study on the Temperature Elevation in Tissue during Moxibustion Therapy

**DOI:** 10.1155/2020/7514302

**Published:** 2020-02-27

**Authors:** Maxim Solovchuk, Hong-An Deng, Tony W. H. Sheu

**Affiliations:** ^1^Institute of Biomedical Engineering and Nanomedicine, National Health Research Institutes, Miaoli 35053, Taiwan; ^2^Department of Engineering Science and Ocean Engineering, National Taiwan University, Taipei 10617, Taiwan; ^3^Center of Advanced Study in Theoretical Sciences (CASTS), National Taiwan University, Taipei 10617, Taiwan

## Abstract

Moxibustion is a thermal therapy in traditional Chinese medicine that relies on the heat from burning moxa to be transferred beneath the skin surface. Although moxibustion has long been in widespread practice, the mechanism of heat transfer modality and temperature distribution during this treatment is not yet well understood. The current paper presents the first examination by magnetic resonance imaging (MRI) of the three-dimensional temperature elevation during moxibustion treatment. A mathematical model for the prediction of temperature elevation during moxibustion therapy has been constructed and compared with the experimental data. Good agreement between the measured temperature and the results of numerical calculations has been found. Tissue up to 3 cm deep can be heated during the treatment. It was revealed that both heat conduction and radiation heat transfer play important roles during the treatment. The results presented in the current paper can be used for understanding the mechanisms of Chinese medicine and developing useful guidelines for Chinese medicine doctors.

## 1. Introduction

Traditional Chinese medicine has a history of thousands of years, since at least its earliest description in the *Yellow Emperor's Inner Classic* in 99 BC. Practically speaking, traditional Chinese medicine includes both herbal remedies and acupuncture. In acupuncture and moxibustion treatments, mechanical and thermal stimulations of acupoints are used to induce biological reactions.

Moxibustion is a thermal therapy in traditional Chinese medicine. Thermal therapy is a widely known therapeutic procedure. Different heating sources can be used, including ultrasonic waves, electromagnetic waves, lasers, and microwaves [1]. Heating tissue up to 41°C is used in physiotherapy and in the treatment of rheumatic diseases [1]. Temperatures of 41–45°C are applied in hyperthermia, with the treatment time ranging from half an hour to several hours. Temperatures higher than 50°C are used for the thermal ablation of tissue in the treatment of cancer and other applications [2–4].

Moxibustion is one of traditional Chinese medicines' main thermal therapeutic modalities; it delivers heat stimulation to specific areas of the body, such as meridian points [5]. Moxibustion is used to treat a wide range of diseases. A bibliometric analysis of papers published from 1954 to 2007 revealed that moxibustion has been reported for treating 364 kinds of diseases [6]. Moxibustion therapy is most commonly applied in cases of malposition, diarrhea, and colitis [7], followed by knee osteoarthritis, temporomandibular joint disturbance syndrome, soft tissue injury, heel pain, asthma, urinary retention, and herpes zoster [7]. Moxibustion and acupuncture rely on thermal and mechanical stimulations of particular points in the human body and inducing different biological reactions [8]. Although moxibustion is a common treatment in Taiwan, China, Japan, and Korea, its mechanisms such as heat transfer modality and temperature distribution are still unclear.

Moxibustion consists of burning dried mugwort (moxa) over different parts of the body. Moxibustion therapy employs both direct moxibustion and indirect moxibustion [9]. In direct moxibustion, the moxa contacts the skin surface directly; and for indirect moxibustion, buffer substances such as ginger, garlic, or salt are placed between the skin and moxa. Air or paper can be used as buffer layers for modern indirect moxa devices. The presence of additional buffer layers gives indirect moxibustion some unique thermal characteristics. As shown by Yi [9], airflow can also influence the treatment. The temperature between the moxa and skin (hot plate) has been measured using a thermocouple. The maximum temperature induced by indirect moxibustion was about 65°C on the skin surface and 45°C in the subcutaneous layer. Different mechanisms may contribute to the treatment, including thermal heat transfer, radiation, and herbal effects of burning moxa [10, 11]. The high temperatures during moxibustion can lead to scars or burns on the skin [12]. It is also quite difficult to quantify the therapeutic effect of moxibustion and the effectiveness of the treatment. Therefore, understanding of the temperature distribution during treatment is very important.

An increase of the temperature in tissue can lead to activation of thermosensitive receptors (TRPV channels), which can induce different biological and chemical reactions in the body [13, 14]. There are multiple transient receptor potential (vanilloid) (TRPV) channels from TRPV1 to TRPV4 that can be activated at different temperatures and in different cells. For example, TRPV1 can be activated at temperatures higher than 42°C, TRPV2 at *T* > 52°C and TRPV3 at *T* > 33°C with different possibilities of opening. Therefore, it is quite important to know the spatial temperature during moxibustion treatment in order to understand moxibustion's mechanisms and the physical, chemical, and biological effects involved in the treatment.

The field of the moxibustion has attracted significant interest in recent years, with both experimental and theoretical studies being performed. Jeon and Choi [15] investigated numerically the temperature elevation during indirect moxibustion treatment by considering unsteady convective heat transfer in the air coupled with conductive heat transfer in tissue. Huang and Sheu [16] studied the effect of moxibustion on blood flow in the leg. The skin-surface temperature during moxibustion treatment has been measured using an infrared camera. Then the obtained experimental data were used as a boundary condition to simulate numerically the temperature beneath the heating position and the heat transfer in blood vessels. In the above studies, it was assumed that heat conduction is the main heating mechanism. In the current study, we will show that radiation heat transfer is also very important and should be taken into account. In Li et al. [11], the authors studied the thermal effect of cigar-shaped moxa. They took into account radiation heat transfer. For the validation of the model, they used only infrared and thermocouple measurements. However, several drawbacks are associated with the use of thermocouples. First, it is difficult to control the position of the thermocouple (positioning error). Second, thermocouples can invasively measure the temperature at just one point. And third, the presence of a thermocouple can increase the heating rate.

MRI permits the measurement of two- or three-dimensional temperature distribution, which can be used to validate the model [17] and does not have the drawbacks mentioned above. In the present work, for the first time MRI temperature measurements are performed and compared with numerical simulation results. The obtained experimental data reveal that radiation heat transfer plays a very important role in moxibustion treatment. Experiments showed that the temperature can be increased in tissue up to 4 cm deep. If only heat conduction is considered, the predicted temperature can be increased only up to 1 cm beneath the skin surface. Very good agreement was found between the experimental and numerical results when radiation heat transfer was included in the mathematical model. The authors believe that the obtained experimental and theoretical results can be helpful for the standardization of therapy procedures and the development of clinical protocols [11, 18].

## 2. Experimental Methods

**Figure 1:**
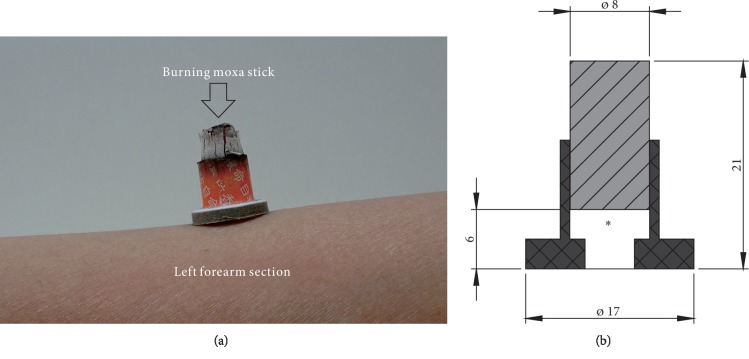
(a) Schematic of the treatment; (b) cross section of the moxa and dimensions of the moxa stick. ∗ denotes the location of a thermocouple during the temperature measurements of air.

A schematic of moxibustion treatment can be seen in Figure 1(a). In the current study, we are going to perform *in vitro* temperature measurements and compare them with the results of numerical simulations. Figure 1(a) shows the schematic of a moxa stick on a slice of pork. The moxa stick is 8 mm in diameter and 15 mm in length, with a weight of 1.7 g. A 6 mm cavity between the moxa and the surface of the skin permits the indirect administration of thermal stimulation, as shown in Figure 1(b). First, the temperature of the air cavity between the moxa and skin will be measured using the thermocouple. The location of the thermocouple can be seen in Figure 1(b). Because ash can be formed during the burning of moxa, we will also study whether ash cleaning can affect the temperature distribution.

There are two mechanisms of the temperature increase from the burning moxa: heat conduction and how it affects the treatment from the skin and radiation heat transfer from the burning moxa. Burning moxa can have a high temperature (c. 548–890°C) [19, 20], and we will show later that both heat conduction and radiation heat transfer play important roles during moxibustion treatment. Since MRI permits 2D and 3D temperature measurements inside the human body, the temperature of tissue beneath the moxa will be measured using an MRI machine.

### 2.1. Moxibustion Temperature Measurement

The *in vitro* tissue moxibustion experiments were performed according to the following procedures. After ignition of a moxa stick, the moxa was placed on the surface of pork and kept burning for 30 min. The temperature tissue was monitored by MRI machine (Symphony, Siemens) in the Institute of Biomedical Engineering and Nanomedicine (I-BEN) of the National Health Research Institutes (NHRI) with the following parameters: TR = 13 ms, TE = 7 ms, flip angle = 30, data matrix 128 × 128, average = 2, field of view (FOV) = 256 × 256 mm, and slice thickness = 5.5 mm. It took 5.0 sec to get one MRI image. Then, image processing software was used to convert the raw data to temperature data by Matlab and Java languages [17].

The schematic of the moxibustion experimental device is shown in Figure 1. The moxa stick was fixed on the porcine tissue and the distance between the moxa and the pork surface was 6 mm. All experiments were performed under the controlled environmental conditions with minimal air flow; the ambient temperature and the relative humidity were kept at the values of 20°C and 1°C and 45% and 55%, respectively. The *in vitro* tissue moxibustion experiments were performed according to the following procedures. The moxibustion lasted for 30 min. The temperature of tissue was measured for 45 min, including the moxibustion process (30 min) and the natural cooling process (15 min).

## 3. Mathematical Model

### 3.1. Biothermal Equations

Heat transfer beneath the skin surface during moxibustion process can be modeled by the Pennes' bioheat equation [21]:

(1) $$\begin{array}{c} \rho_{t}c_{t}\frac{\partial T}{\partial t}=\nabla \cdot \left(k_{t}\nabla T\right)+\rho_{b}c_{b}\omega_{b}\left(T_{0}-T\right)+Q_{\text{met}}+Q, \end{array}$$

where *ρ*_*t*_ is the density of tissue (kg/m^3^), *c*_*t*_ the specific heat of tissue (J/kg·K), *k*_*t*_ the thermal conductivity of tissue (W/m·K), *ρ*_*b*_ the density of blood (kg/m^3^), *c*_*b*_ the specific heat of blood (J/kg·K), *ω*_*b*_ the blood perfusion rate (s^−1^), *Q*_met_ the volumetric metabolic heat (W/m^3^), and *Q* the heat source term (W/m^3^). In the current paper, *ex vivo* experiments have been performed; therefore *Q*_met_ and *ω*_*b*_ are both zero. For the modeling of the heat transfer in a slice of pork, the above equation can therefore be reduced to the following partial differential equation:

(2) $$\begin{array}{c} \rho_{t}c_{t}\frac{\partial T}{\partial t}=\nabla \cdot \left(k_{t}\nabla T\right)+Q, \end{array}$$

where *Q* is the radiation heat source released by burning moxa stick (W/m^3^). The thermal properties of tissue are listed in Table 1 [17].

### 3.2. Radiation Heat Transfer

In the current paper, the temperature increase inside the porcine muscle during moxibustion treatment was measured using MRI [16]. Previously, temperature on the skin surface has been measured with an infrared camera [8]. In the current paper, both data sets for temperature on the surface have been used as a boundary condition in the simulation of the heat equation (2). Burning moxa emits visible light and infrared radiation; therefore, besides the heat effects, nonthermal radiation effects may have an important role in the efficacy of moxibustion. Physics tells us that radiation is a process of energy transfer in the form of electromagnetic waves; any object with a temperature above absolute zero emits electromagnetic radiation. The [22] infrared radiation spectrum of the moxa stick was 1.5–14 *μ*m.

Since a burning moxa stick has a very high temperature, it is also necessary to take the effect of radiation heat transfer from the burning moxa into account. As a result, the heat source term *Q* in equation (1) includes radiation attenuation in the tissue. The radiation energy from burning moxa stick is absorbed and scattered when it transmits through the tissue. The intensity of radiation decreases exponentially with depth based on Beer's Law: exp(−|*y*|/*μ*_*a*_), where *y* is the depth and *μ*_*a*_ is the penetration depth (cm). The absorbed radiation energy in tissue *Q* can be described by

(3) $$\begin{array}{c} Q=Q_{\text{surface}}e^{-\left(\left|y\right||/|\mu_{a}\right)}, \end{array}$$

where *Q*_surface_ (W/m^3^) represents the heat source on the surface of the pork. For the porcine muscle, *μ*_*a*_=0.015 m [11]. *Q*_surface_ has been adjusted based on the experimental data on the surface of the pork.

**Figure 2:**
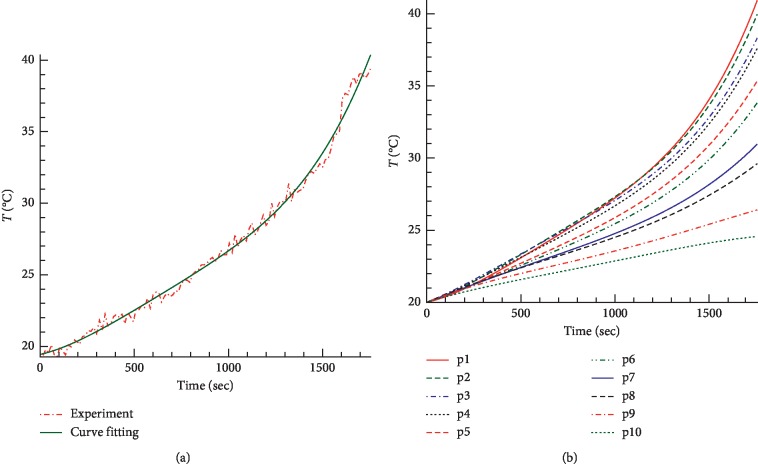
Temperature as a function of time at the heating position on the pork surface: (a) p1 curve fitting data; (b) the temperature curves fitted from experimental data by the least squares method.

The temperature on the pork surface during the experiment was controlled to be lower than 60°C, which is the highest temperature that human skin can endure and without scarring.  Figure 2 shows the measured temperature at different points on the pork surface as a function of time.

### 3.3. Numerical Methods

The initial temperature of the moxa stick and ambient temperature are set according to the experimental data. The ambient temperature is assumed to be *T*_*a*_ = 20°C.

The simulation of the temperature increase during moxibustion has been performed with the finite volume method. Mesh independence test was carried out by comparing the temperature in the final working mesh with the temperature obtained in a refined mesh, which was generated by increasing the number of cells by 50%. In these two meshes, the temperatures differ from each other by less than 1%. A time step of 0.5 sec was decided upon by taking the computational convergence and efficiency into account. The treatment time was 30 min, followed by natural cooling of about 15 min.

## 4. Results and Discussion

Burning moxa stick without smoke can produce a high temperature, about 548–890°C [19, 20]. It renders a warm feeling when it is close to the body, so the treatment has an essential thermal physical effect [23]. To avoid scars, it is better to keep the maximum temperature on the surface below 60°C. The actual temperature of indirect moxibustion is greatly affected by the texture, size, and moisture content of the insulating material [9]. The environment can also affect the treatment. Yi [9] found that airflow can affect the treatment duration and maximum temperature.

**Figure 3:**
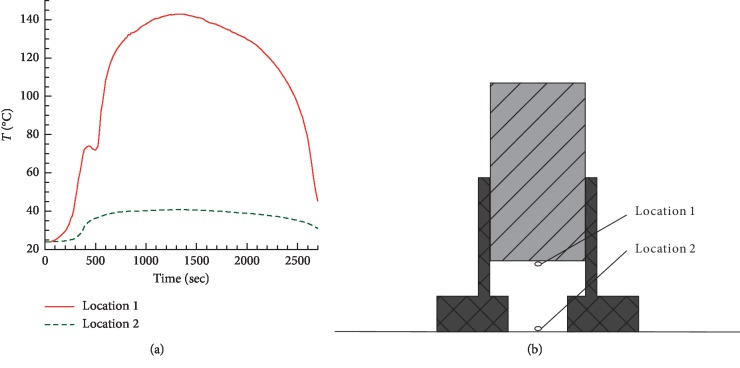
(a) Temperature at two points, as measured by thermocouples during moxibustion treatment without ash cleaning; (b) location of the two points where the temperature was measured.

In Figure 3 the measured temperature of air between the moxa and skin is presented as a function of time. The measurements at two points (close to the moxa and close to the skin) were performed using thermocouples. The moxibustion lasted for 30 min, followed by 15 min of cooling. The temperature of air close to the pork surface reached the maximum value of about 135°C at time 24–25 min. Similarly, a peak temperature about 130°C was measured by Yi [9]. The maximum temperature on the surface of pork was about 40°C. Since thermocouples allow measurement of temperature only at a single point, we made use of MRI in the remaining sections of the manuscript for volumetric temperature measurements in tissue.

### 4.1. The Effect of Radiation Heat Transfer on the Temperature Increase in Tissue

**Figure 4:**
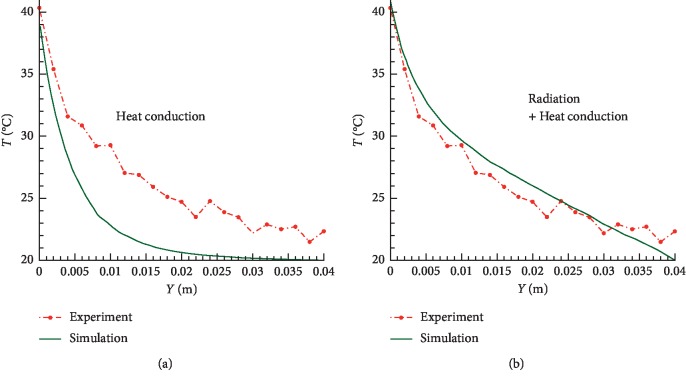
The measured (MRI) and computed temperature profiles in pork along the *y*-axis at time *t* = 1755 sec. (a) Only heat conduction has been considered in the simulations; (b) both heat conduction and radiation were considered during the modeling of moxibustion.

The predicted and measured temperature profiles as the functions of depth are presented in Figure 4. In most previous theoretical studies of the temperature elevation in tissue during moxibustion treatment [8, 15], only the heat conduction effect was considered; it was concluded [8, 15] that only a thin layer close to the skin (about 1 cm in depth) can be heated. However, as shown in Figure 4(a), if only heat conduction is considered in the simulation, the predicted results underestimate the real temperature. Experimental results show that even deep tissues up to 4 cm in depth can be heated (Figure 4(a)). Burning moxa can have a high temperature (around 548–890°C) [6, 20]. Therefore, it is very important to take into account radiation heat transfer.

After including the radiation heat transfer effect (Figure 4(b)), good agreement between experimental and numerical simulation results was found. Although there is no direct interaction between the flame and skin (or slice of pork), radiation still plays an important role in moxibustion treatment.

### 4.2. Experiment and Simulation Results

**Figure 5:**
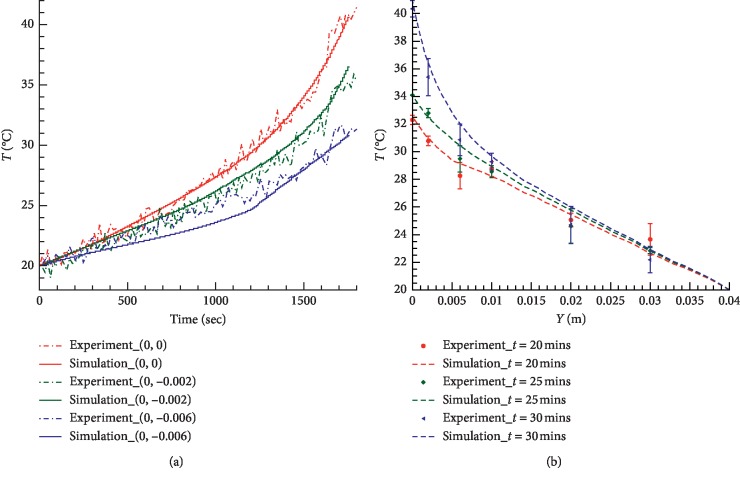
The measured and predicted temperatures at monitoring points in *in vitro* tissue (a) as the function of time at different depths, *h* = 0, *h* = 0.002 m, and *h* = 0.006 m, and (b) as the function of depth at *t* = 20 min, 25 min, and 30 min.

**Figure 6:**
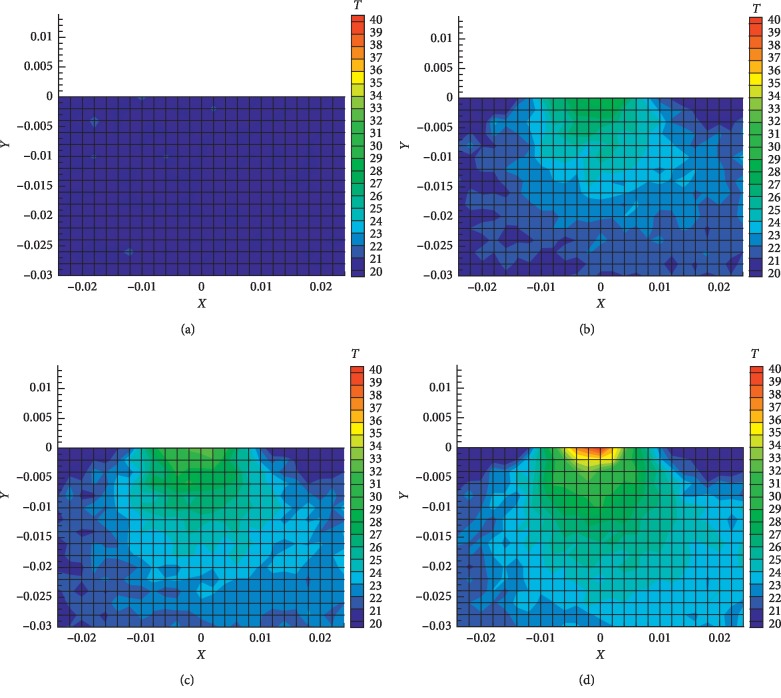
The measured temperature profiles in pork at the plane perpendicular to the surface at different time intervals: (a) *t* = 0 sec; (b) *t* = 1200 sec; (c) *t* = 1500 sec; (d) *t* = 1755 sec.

The thermal distribution of burning moxa stick was studied experimentally and in a mathematical simulation. In Figure 5(a), one can see that the predicted and measured temperatures at the monitoring points (0, 0) and (0, −2) mm are in a good agreement. The deviation between the simulated and experimental results at the monitoring point (0, −6) mm may be attributed to the inhomogeneity and anisotropy of biological tissue. It can be seen from Figure 5 that our predictions of the moxibustion treatment are all in agreement with the measured temperatures in general. In Figure 6 the measured temperature profiles in pork at different time steps are presented at the slice, perpendicular to the surface.

**Figure 7:**
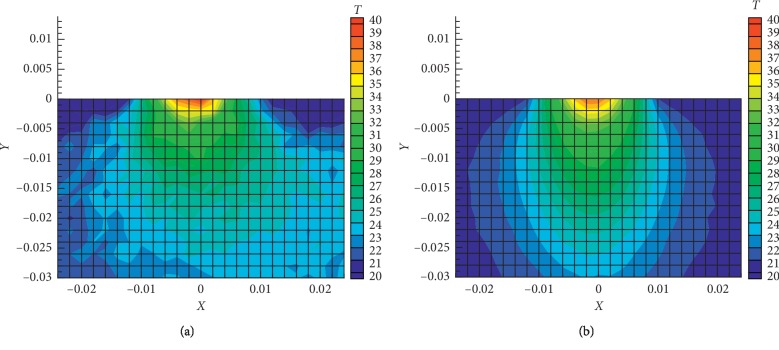
The (a) measured and (b) simulated temperature profiles in pork at the plane perpendicular to the surface at time *t* = 1755 sec.

**Figure 8:**
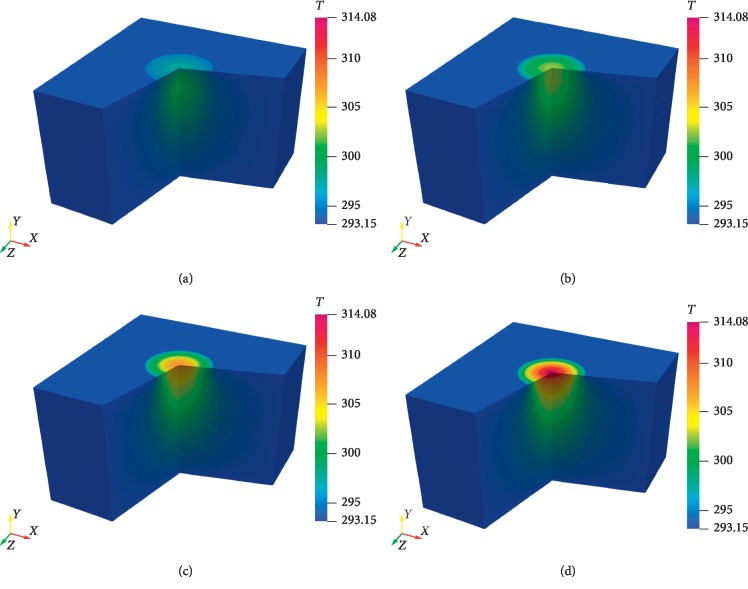
The simulated temperature distribution in porcine muscle at different time intervals: (a) *t* = 600 sec; (b) *t* = 1200 sec; (c) *t* = 1500 sec; (d) *t* = 1755 sec.

In Figure 7 the predicted and measured two-dimensional temperature contours are presented at the cutting plane perpendicular to the surface of pork. Good agreement between the predicted and measured temperatures can be seen. The predicted three-dimensional temperature distribution at different time steps is presented in Figure 8.

In the current study, the temperature beneath the skin during moxibustion treatment was measured using MRI. From the experimental data, it can be seen that tissues up to 3-4 cm deep can be heated. Physiological studies have shown different heat responses in sensory fibres [13]. Some fibres respond to the moderate threshold (43°C) and very few respond to higher temperature (52°C). Heat-activated signal transduction usually occurs in the nerves at skin level. However, the signalling molecules sensitive to temperature are also present in peripheral nervous system (PNS), brain, endothelium, connective tissue, and different organs (e.g., kidney, liver, and heart). These signalling molecules after thermal activation can induce different biological and chemical reactions in the body [13, 24]. Transient receptor potential (TRP) ion channels activated by temperature (thermo-TRPs) are important molecular players in acute, inflammatory, and chronic pain states [13, 14, 25], where moxibustion treatment is very efficient.

Temperature-activated transient receptor potential ion channels (thermo-TRPs) can be activated at different temperatures and with different possibilities [13], as it can be seen in Table 2. Let's assume that a temperature increase of about 5°C in human body can activate TRPV channels (which can be true for trpv1, trpv3, and trpv4) and consider this temperature as an approximate threshold value. As can be seen in experimental and numerical results, tissues up to 2.5 cm beneath the skin can be heated up to 5°C during moxibustion. It means that the thermosensitive channels can be activated within an approximate depth of 2.5 cm below the skin surface (for deeper tissue, the possibility of activation will be lower). Further research is necessary to gain a better understanding of these processes. In the current study, *ex vivo* experiments have been performed. For *in vivo* experiments, the results may slightly vary due to tissue inhomogeneity and blood perfusion [4, 8]. The authors believe that the obtained results can help deepen understanding of the mechanisms involved in thermal therapy. In future studies we will perform *in vivo* (animal and human) experiments.

### 4.3. The Effect of Ash Cleaning

The temperature of air between the moxa and skin during moxibustion treatment was measured. Thermocouples were used for the measurements of the air temperature. The exact location of the measurement point was as shown in Figure 1(b). Ash formation can slow down the burning of moxa. Therefore, it was also decided to check whether ash cleaning can affect the treatment.

**Figure 9:**
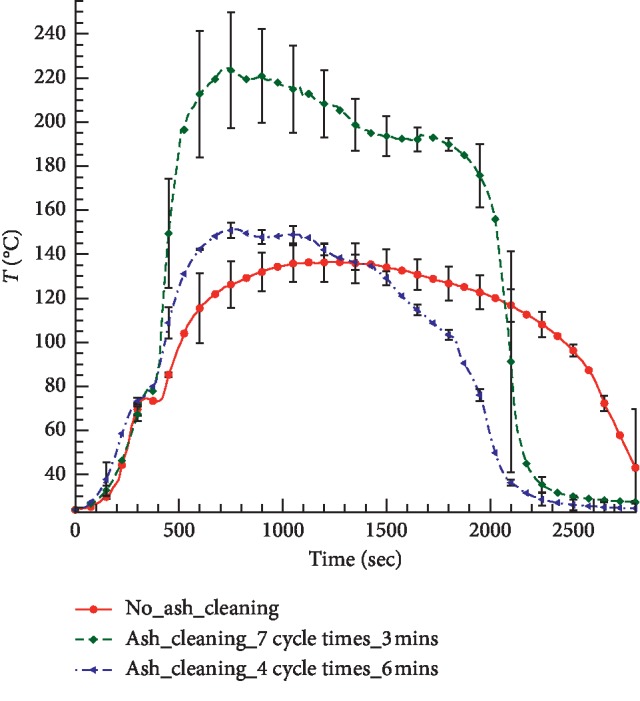
The measured temperature of air between the moxa and pork with and without ash cleaning. The locations of the points at which the temperature measurements were performed can be seen in Figure 1.

In Figure 9 the temperature of air close to the pork surface is presented as a function of time. Three different cases were considered: without ash cleaning, ash cleaned every 3 min, and ash cleaned every 6 min. Every experiment was performed 3–5 times; the average data are plotted in Figure 9, together with the experimental error.

Experiments confirmed that during moxibustion the temperature of the air close to the surface of the pork can reach a maximum value of about 137°C without ash cleaning. With ash cleaning, the temperature increases immediately after the ash is removed. The maximum temperature becomes higher and the burning process becomes shorter compared with the case without ash cleaning. The maximum average temperature with an ash-cleaning cycle of 3 min was almost 74°C higher than that with an ash-cleaning cycle of 6 min. These results suggest that ash cleaning can accelerate burning of moxa stick and produce a relatively high temperature during treatment. In Sun et al. [26], similar conclusions were drawn. The authors considered the cigar-shaped moxa, which was located 3 cm from the skin. Since for the cigar-shaped moxa the flame is directed toward the skin, the effect of ash cleaning was more pronounced, and some temperature oscillations were observed. Doctors should consider these effects during moxibustion treatment.

## 5. Conclusions

In the present study, for the first time the three-dimensional temperature distributions during moxibustion experiment on biological tissue have been measured. Most previous studies took only infrared measurements of skin temperature or used only thermocouple measurements [8, 9, 11]. Thermocouples allow temperature measurement at a single point. In the current paper, MRI temperature measurements were performed. MRI can noninvasively measure three-dimensional temperature beneath the skin, which has not been reported before. It was shown that even for a depth of about 3-4 cm, there was still a significant increase in temperature. The measured temperature increase can't be explained by only heat conduction. Radiation heat transfer plays a significant role as well during moxibustion.

A mathematical model for the prediction of temperature elevation during moxibustion treatment has been proposed. The measured temperature increase has been compared with the numerical simulation results and good agreement has been found. The results of the current study could reveal the underlying mechanism of moxibustion therapy regarding the transfer of heat through not only conduction but also radiation.

Ash cleaning is an effective way to keep the temperature of the moxa stick high. A shorter ash-cleaning cycle can generate a higher temperature range. Ash deposits on moxa sticks have an adverse effect on moxibustion performance. It was found that a shorter interval between ash-cleaning cycles led to a higher temperature on the tissue surface.

Our studies have focused mainly on the primary factors that affect the thermal characteristics of moxibustion. These are essential to achieve better therapeutic results from moxibustion. The simulated results can be useful to understand the mechanisms involved in moxibustion therapy and to develop an effective guideline on moxibustion for Chinese medicine doctors.

## Figures and Tables

**Table 1:** Thermal properties for the porcine muscle and water.

Tissue	*c*_0_ (m/s)	*ρ* (kg/m^3^)	*c* (J/kg·K)	*k* (W/m·K)
Muscle	1550	1055	3200	0.51
Water	1520	1000	4200	0.6

**Table 2:** Properties of thermosensitive channels [13].

Name	Temperature sensitivity (°C)
Trpv1	≥42
Trpv2	≥52
Trpv3	>33
Trpv4	∼27–42

## Data Availability

All the data used to support the findings of this study have been included in the paper.
